# Draft of a Bill to Amend the Law Relating to Dental Practitioners in Great Britain

**Published:** 1878-03

**Authors:** 


### DRAFT OF A BILL TO AMEND THE LAW RELATING TO DENTAL PRACTITIONERS IN GREAT BRITAIN.

WHEREAS it is expedient that provision be made for the registration of persons specially qualified to practice as Dentists in the United Kingdom, and that the law relating to persons practicing as Dentists be otherwise amended:

Be it therefore enacted

BY THE QUEEN'S MOST EXCELLENT MAJESTY, by and with the advice and consent of the Lords Spiritual and Temporal, and Commons in this present Parliament assembled, and by the authority of the same, as follows, (that is to say):—

1. This Act may for all purposes be cited as the Dentist's Act, 1878.

2. In this Act "General Council" means the General Council of Medical Education and registration of the United Kingdom, established under the Medical Act, 1858; and "Branch Council" means a branch of said Council as constituted by the same Act:

"Registrar" means a person appointed to be a Registrar by a General Council or a Branch Council under the Medical Act, 1858.

### REGISTRATION.

3. From and after the *First* day of *August*, 1879, a person shall not be entitled to take or use the name or title of "Dentist" (either alone or in combination with the word "Surgeon" or with any other word or words), "Dental Surgeon," or "Dental Practitioner," or any name title addition or description implying that he is registered under this Act or that he is a person specially qualified to practise Dentistry, unless he is registered under this Act.

Any person who, after the *First* day of *August*, 1879, not being registered under this Act, takes or uses any such name title addition or description as aforesaid, shall be liable, on summary conviction, to a fine not exceeding *Twenty pounds*.

4. From and after the *First* day of *August*, 1879 a person shall not be entitled to recover any fee or charge, in any Court, for the performance of any dental operation or for any dental attendance or advice, unless he is registered under this Act or is a legally qualified medical practitioner.

5. Any person who—

(*a*) Is a Licentiate in Dental Surgery or Dentistry, of any Royal College of Surgeons in the United Kingdom, or of the Faculty of Physicians and Surgeons of Glasgow; or,

(*b*) Is at the passing of this Act bona fide engaged in the practice of dentistry, either separately or in conjunction with the practice of medicine or surgery, shall be entitled to be registered under this Act: Provided that a person shall not be registered under this Act as having been at the passing thereof engaged in the practice of dentistry unless he produces or transmits to a Registrar, before the *First* day of *August*, 1879, information of his name and address, and a declaration signed by him in the form in the schedule to this Act or to the like effect; and the Registrar may, if he sees fit, require the truth of such declaration to be affirmed in manner provided by the Act of the Session held in the fifth and sixth years of the reign of King William the Fourth, chapter sixty-two, intituled "*An Act to repeal an Act of the present Session of Parliament, intituled An Act for the more effectual Abolition of Oaths and Affirmations taken and made in various Departments of the State, and to substitute Declarations in lieu thereof, and for the more entire Suppression of voluntary and extra judical Oaths and Affidavits; and to make other Provisions for the Abolition of unnecessary Oaths.*"

A person resident in the United Kingdom shall not be disqualified for being registered under this section by reason that he is not a British subject; and a British subject shall not be disqualified for being registered under this section by reason of his being resident beyond the limits of the United Kingdom.

6. It shall be the duty of the Registrars to make and keep for England, Scotland, and Ireland respectively local registers, and to enter therein the names and addresses and the qualifications of persons entitled and applying to be registered under this Act.

The Registrars shall keep such registers correct in accordance with the provisions of this Act and the orders and regulations of the General Council to be made under this Act, and shall from time to time erase the names of all registered persons who have died, and make the necessary alterations in the addresses or qualifications of registered persons.

Any Registrar may address a letter (to be forwarded by post as a registered letter according to the Post Office regulations for the time being in force) to any registered person according to his address on the register, inquiring whether he has ceased to practice, or has changed his residence; and if no answer is returned to such letter within six months from the sending of the letter, a second of similar purport shall be sent in like manner; and if no answer is returned to such letter within three months from the sending thereof, it shall be lawful to erase the name of such person from the register: Provided that a name so erased may at any time be restored by direction of the General Council.

7. No name shall be entered in a register under this Act except of persons authorised by this Act to be registered, nor unless a Registrar be satisfied by sufficient evidence that the person claiming is entitled to be registered; any person aggrieved by the refusal of a Registrar to enter his name may appeal to the General Council, who shall hear and determine the appeal in such manner as they see fit; and any entry which is at any time proved to the satisfaction of the General Council or of a Branch Council, to have been fraudulently or incorrectly made may be erased from or amended in the register by direction of such General Council or Branch Council.

8. The Registrar of the General Council shall cause all entries and alterations entered and made in the local registers under this Act to be entered and made in a general register to be called the "Register of Dentists," and shall, in the month of January in every year, cause to be printed, published, and sold, under the direction of such Council, correct copies of such register as existing on the Thirty-first day of December last preceding.

9. The Registrars of the Branch Councils for Scotland and Ireland shall with all convenient speed send to the Registrar of the General Council a copy, certified under their hands respectively, of each entry or alteration entered or made by them under this Act, for the purpose of being entered or made in the general register; and the entries in the general register shall bear date from the local registers.

10. The Registrars of the Branch Councils may demand and receive, in respect of the registration, of any person who, before the first day of January 1879, applies to be registered under this act, a fee not exceeding two pounds; and in respect of the registration of any person who after that day applies to be registered, a fee not exeeeding five pounds.

11. The General Council may, if they see fit, establish in the Register of the Déntists distinct sections for the registration of persons (not being British subjects) resident in the United Kingdom, and possessing such foreign qualifications as in the opinion of the Council are a sufficient guarantee of the possession of the requisite knowledge and skill for the efficient practice of dentistry, and for the registration of persons so resident and possessing such colonial qualifications as in the opinion of the Council are such guarantee as aforesaid.

Any person registered in either of such sections shall be deemed for all purposes to be registered under this act.

12. Subject to the provisions of this act, the General Council may from time to time make, alter, and revoke such orders and regulations as they see fit for regulating the general register and the local registers, and the practice of registration under this act, and the fees to be paid in respect thereof.

EXAMINATIONS.

13. Notwithstanding anything in any Act of Parliament, charter, or other document, it shall be lawful for the Royal College of Surgeons of Edinburgh, and the Faculty of Physicians and Surgeons of Glasgow, and the Royal College of Surgeons in Ireland, from time to time to hold examinations for the purpose of testing the fitness of persons to practice as dentists who may be desirous of being so examined, and to grant certificates of such fitness, and any person who obtains such a certificate from any of those colleges or bodies, shall be a licentiate in dental surgery or dentistry of such college or body, and his name shall be entered on a list of such licentiates to be kept by such college or body.

Each of the said colleges or bodies shall admit to the examinations held by them respectively under this section any person desirous of being examined who has attained the age of twenty-one years, and has complied with the regulations in force (if any) as to education of such college or body.

14. The Council or other the governing body of the Royal College of Surgeons of Edinburgh, and the Faculty of Physicians and Surgeons of Glasgow, and the Royal College of Surgeons in Ireland respectively, may from time to time appoint a board of examiners for the purpose of conducting the examinations and granting the certificates hereinbefore mentioned.

Each of such boards shall be called the Board of Examiners in Dental Surgery or Dentistry, and shall consist of not less than six members, one-half of whom at least shall be persons registered under this act, and such registration shall (notwithstanding anything in any Act of Parliament, charter or other document) be deemed the only qualification necessary for the membership of such board.

The persons appointed by each such council or other governing body shall continue in office for such period, and shall conduct the examinations in such manner, and shall grant certificates in such form as such council or other governing body may from time to time, by by-laws or regulations, respectively direct.

A casual vacancy in any such board of examiners may be filled by the council or other governing body which appointed such board, but the person so appointed shall be qualified, as the person in whose stead he is appointed was qualified, and shall hold office for such time only as the person in whose stead he is appointed would have held office

15. Such reasonable fees shall be paid for the certificate to be granted under this Act by the Board of Examiners of the Royal College of Surgeons of Edinburgh, the Faculty of Physicians and Surgeons of Glasgow, and the Royal College of Surgeons in Ireland respectively as the council or other governing body of each of those colleges or bodies may from time to time, by by-laws or regulations, respectively direct.

16. The Royal College of Surgeons of England, shall continue to hold examinations and to appoint a board of examiners in dental surgery for the purpose of testing the fitness of persons to practice as dentists who may be desirous of being so examined, and to grant certificates of such fitness, subject and according to the provisions of their charter dated the eighth day of September 1859, and the by-laws made, or to be made, in pursuance thereof; and any person who obtains such a certificate shall be a licentiate in dental surgery of the said college, and his name shall be entered on a list of such licentiates to be kept by the said college.

17. The Royal College of Surgeons, of England, and the Royal College of Surgeons of Edinburgh, and the Faculty of Physicians and Surgeons of Glasgow, and the Royal College of Surgeons in Ireland respectively shall from time to time, when required by the General Council, furnish such council with such information as such council may require as to the course of study and examinations to be gone through, in order to obtain such certificates as are in this act mentioned, and generally as to the requisites for obtaining such certificates; and any member or members of the General Council, or any person or persons deputed for this purpose by such council, or by any Branch Council, may attend and be present at any such examination.

18. The Royal College of Surgeons of England, and the Royal College of Surgeons of Edinburgh, and the Faculty of Physicians and Surgeons of Glasgow, and the Royal College of Surgeons in Ireland, or any two or more of them, may, with the sanction and under the direction of the General Council, unite or co-operate in conducting the examinations for certificates under this Act.

19. Where it appears to the General Council that the course of study and examinations to be gone through, in order to obtain such certificate as in this act mentioned from any of the said colleges or bodies are not such as to secure the possession by persons obtaining such certificate of the requisite knowledge and skill for the practice of dentistry, the General Council may represent the same to Her Majesty's Privy Council.

20. The Privy Council, on any representation made as aforesaid, may, if they see fit, order that a certificate granted by any such college or body after such time as may be mentioned in the order shall not confer any right to be registered under this Act.

Any such order may be revoked by the Privy Council on its being made to appear to them, by further representation from the General Council or otherwise, that such college or body has made effectual provision, to the satisfaction of the General Council, for the improvement of such course of study or examination.

The powers by this Act vested in the Privy Council may be exercised by any three or more of the Lords and others of the Privy Council, the Vice-President of the Committee of the said Privy Council on Education being one of them.

21. After the time mentioned in this behalf in any such Order in Council, no person shall be entitled to be registered under this Act in respect of a certificate granted by the college or body to which such order relates after the time therein mentioned, and the revocation of any such order shall not entitle any person to be registered in respect of a certificate granted before such revocation.

22. A certificate under this Act shall not confer any right or title to be registered under the Medical Act, 1858, in respect of such certificate, nor to assume any name, title, or designation implying that the person mentioned in the certificate is by law recognised as a licentiate or practitioner in medicine or general surgery.

### SUPPLEMENTAL.

23. A copy of the Register of Dentists for the time being, purporting to be printed and published in pursuance of this Act, shall be evidence in all Courts (until the contrary be made to appear) that the persons therein specified are registered according to the provisions of this Act; and the absence of the name of any person from such copy shall be evidence (until the contrary be made to appear) that such person is not registered according to the provisions of this Act: Provided that, in the case of any person whose name does not appear in such copy, a certified copy under the hand of the Registrar of the General Council or of any Branch

Council of the entry of the name of such person on the general or on a local register shall be evidence that such person is registered according to the provisions of this Act.

24. Every person registered under this Act shall be exempt, if he so desires, from serving on all juries and inquests whatsoever, and from serving all corporate parochial ward hundred and township offices, and from serving in the militia; and the name of any registered person shall not be returned in any list of persons liable to serve in the militia, or in any such office as aforesaid.

25. All moneys arising from fees paid on registration or from the sale of copies of the registers under this Act shall be applied in defraying the expenses of registration and the other expenses of the execution of this Act, in accordance with such regulations as may be from time to time made by the General Council.

26. The Treasurers of the General and Branch Councils shall enter in books, to be kept for that purpose, a true account of all sums of money by them received and paid under this Act; and such accounts shall be submitted by them to the General Council and Branch Councils respectively at such times as the Councils may respectively require. Such accounts shall be published annually; and shall be laid before both Houses of Parliament in the month of March in every year if Parliament be then sitting; or, if Parliament be not sitting, then within one month after the commencement of the next setting of Parliament.

27. Any registrar who wilfully makes or causes to be made any falsification in any matter relating to any register under this Act shall be deemed guilty of a misdemeanour in England or Ireland, and in Scotland of a crime or offence punishable by fine or imprisonment, and shall, on conviction thereof, be liable to be imprisoned for any term not exceeding twelve months.

28. Any person who wilfully procures or attempts to procure himself to be registered under this Act, by making or producing or causing to be made or produced any false or fraudulent representation or declaration, either verbally or in writing, and any person aiding and assisting him therein, shall be deemed guilty of a misdemeanour in England and Ireland, and in Scotland of a crime or offence punishable by fine or imprisonment, and shall, on conviction thereof, be liable to be imprisoned for any term not exceeding twelve months.

29. If any person registered under this act is convicted in England or Ireland of any felony or misdemeanor, or in Scotland of any crime or offense; or, after due inquiry, is jugded by the General Council to have been guilty of infamous conduct in any professional respect, the General Council may, if they see fit, direct the name of such person to be erased from the register of registers on which it appears; and any name so erased shall also be erased from the list of licentiates in dental surgery or dentistry of the college or body of which such person is a licentiate.

30. Every Registrar of deaths in the United Kingdom, on receiving notice of the death of any person registered under this act, shall forthwith transmit by post, to the Registrar of the General Council and to the Registrar of the Branch Council for that part of the United Kingdom in which the death occurs, a certificate under his own hand of such death, with the particulars of time and place of death, and may charge the cost of such certificate and transmission as an expense of his office.

31. It shall be lawful for the General Council by special order to dispense with such provisions of this act, or with such part of any by-laws, orders, or regulations made by its authority, as to them may seem fit, in favor of dental students or apprentices who have commenced their professional education or apprenticeship before the passing of this act.

Mar-3

32. All by-laws, orders and regulations made by the General Council or by any college or body under the authority of this act, shall be made and may be from time to time altered or revoked in such manner, and subject to such approval or confirmation (if any), as in the case of other by-laws, orders, or regulations made by such college or body.

33. All fees and penalties under this act, or under any by-law made in pursuance of this act, may be recovered and enforced as follows, that is to say:—In England before two or more justices of the peace in manner directed by the act of the session of the eleventh and twelfth years of the reign of Her present Majesty, chapter fourty-three, entituled "An act to facilitate the performance of the duties of justices of the peace out of sessions within England and Wales with respect to summary convictions and orders," and any act amending the same: and in Scotland before the sheriff or two justices in manner provided by the "Summary Procedure act, 1864:" And in Ireland, within the police district of Dublin metropolis, in manner directed by the acts regulating the powers and duties of justices of the peace for such district or of the police of such district, and elsewhere in Ireland before two or more justices of the peace in manner directed by the "Petty Sessions (Ireland) act, 1851," and any act amending the same.

